# Increased circulating galectin-1 levels are associated with the progression of kidney function decline in patients undergoing coronary angiography

**DOI:** 10.1038/s41598-020-58132-1

**Published:** 2020-01-29

**Authors:** Chin-Sung Kuo, Ruey-Hsing Chou, Ya-Wen Lu, Yi-Lin Tsai, Po-Hsun Huang, Shing-Jong Lin

**Affiliations:** 1 Division of Endocrinology and Metabolism, Department of Medicine, Taipei Veterans General Hospital, Taipei, Taiwan; 2 Cardiovascular Research Center, National Yang-Ming University, Taipei, Taiwan; 3 Institute of Clinical Medicine, National Yang-Ming University, Taipei, Taiwan; 4 Division of Cardiology, Department of Medicine, Taipei Veterans General Hospital, Taipei, Taiwan; 5 Department of Critical Care Medicine, Taipei Veterans General Hospital, Taipei, Taiwan; 6 Healthcare and Services Center, Taipei Veterans General Hospital, Taipei, Taiwan; 7 Taipei Heart Institute, Taipei Medical University, Taipei, Taiwan

**Keywords:** Predictive markers, Interventional cardiology, End-stage renal disease

## Abstract

Galectin-1 modulates acute and chronic inflammation, and is associated with glucose homeostasis and chronic renal disease. Whether the serum galectin-1 level can predict short-term and long-term renal outcomes after contrast exposure in patients undergoing coronary angiography (CAG) remains uncertain. This study aimed to evaluate the relationship between the serum galectin-1 level and the incidence of contrast-induced nephropathy (CIN), and to investigate the predictive role of the circulating galectin-1 level for renal function decline in patients undergoing CAG. In total, 798 patients who had undergone CAG were enrolled. Baseline creatinine and serum galectin-1 levels were determined before CAG. CIN was defined as an increase in the serum creatinine level of 0.5 mg/dl or a 25% increase from baseline within 48 h after the procedure, and renal function decline was defined as > 30% reduction of the estimated glomerular filtration rate from baseline. All patients were followed for at least 1 year or until the occurrence of death after CAG. Overall, CIN occurred in 41 (5.1%) patients. During a median follow-up period of 1.4 ± 1.1 years, 80 (10.0%) cases showed subsequent renal function decline. After adjustment for demographic characteristics, kidney function, traditional risk factors, and medications, higher galectin-1 levels were found to be associated independently with a greater risk of renal function decline [tertile 2: hazard ratio (HR) 5.56, 95% confidence interval (CI) 1.79–17.22; tertile 3: HR 5.56, 95% CI 1.97–16.32], but not with CIN, regardless of the presence of diabetes. In conclusion, higher baseline serum galectin-1 levels were associated with a greater risk of renal function decline in patients undergoing CAG, but were not associated independently with CIN.

## Introduction

Chronic kidney disease (CKD) is a serious public health problem, and its incidence and prevalence are increasing^1^. The early detection of individuals at risk of CKD development or progression is especially important, as early-stage CKD is prevalent and contributes greatly to cardiovascular disease. Research clearly indicates that patients with coronary artery disease (CAD) are at increased risk of progressive renal dysfunction^2^. These patients may be further exposed to the risk of contrast-induced nephropathy (CIN), which has an incidence rate of 4.4–22.1%, if they undergo coronary angiography (CAG)^3^. CIN has traditionally been considered to be a benign and reversible disease, but it may prolong hospital stays and increase in-hospital mortality^4^. Accumulating evidence suggests that CIN is related to long-term renal function decline, in addition to short-term outcomes^5,6^. Efforts to predict the occurrence of CIN include the development of risk scoring systems composed of baseline risk factors^7^, renal Doppler evaluation^8^, 24-h serum creatinine elevation^9^, and renal injury biomarkers^10–14^. However, no predictive marker for long-term renal outcomes in patients undergoing CAG has been established.

Lectin–glycan interactions have been reported to be regulators of extensive physiological and pathological processes. Galectins form a group of proteins that can bind to β-galactoside sugars by N- or O-linked glycosylation through their carbohydrate recognition domains^15^. Galectins comprise three categories: monomeric, chimeric, and tandem-repeat types. They are characterized as homodimers of 14-kDa subunits with two β-galactoside–binding sites, and are expressed in many tissues^15^. Fifteen galectin isoforms, numbered in order of discovery (galectin-1–15), are recognized^15^. Galectin-1 is found in cytoplasm and on cell surfaces, and can be secreted to the extracellular matrix^15^. It modulates cell signaling, proliferation, and survival, and contributes to the control of inflammation and neovascularization^16^.

Diabetes is a traditional risk factor for CIN^7^ and CKD^17^. Recent evidence indicates that galectin-1 levels are increased in the proteome in subcutaneous interstitial fluid^18^ and plasma^19^ in patients with type 2 diabetes. The galectin-1 level is also elevated in the glomerular proteome in mice with CKD^20^. An *in vitro* study implicated galectin-1 in diabetic nephropathy, as seen in human podocytes in high-glucose culture^21^. Furthermore, galectin-1 was reported to be a new fibrosis protein and potential treatment target in the context of diabetic nephropathy^22^. However, whether the serum galectin-1 level can predict CIN and kidney function decline in patients undergoing CAG remains unclear. This study aimed to evaluate the relationship between the serum galectin-1 level and the incidence of CIN, and to clarify the predictive role of galectin-1 in renal function decline, in patients with suspected CAD undergoing CAG at a single hospital. We hypothesized that elevated galectin-1 levels, which have been reported to be associated with diabetes and diabetic nephropathy, would also be predictive markers of CIN and renal function decline in these patients. We also sought to clarify whether the possible predictive role of galectin-1 would be independent of diabetes.

## Results

### Baseline patient characteristics

**Figure 1:**
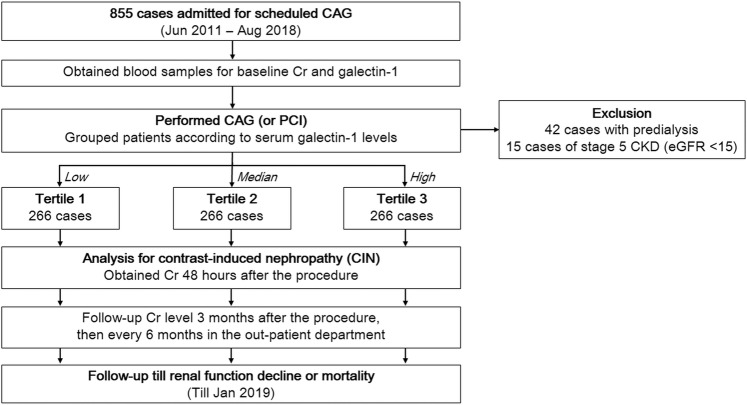
Flowchart of patient recruitment and follow-up.

In total, 798 subjects who underwent elective CAG and/or percutaneous coronary intervention (PCI) were enrolled in this analysis (Fig. 1). The mean age of the study population was 67 ± 12 years, and 67.5% of patients were male. Table 1 summarizes the clinical and demographic characteristics of the patients, grouped by galectin-1 concentration. Patients with higher plasma galectin-1 concentrations were older and had higher incidences of hypertension, diabetes, CKD, heart failure, and multiple vessel disease. Subjects in the highest galectin-1 tertile had significantly increased levels of fasting glucose, proteinuria, and decreased hemoglobin levels, estimated glomerular filtration rates (eGFRs), and left ventricular ejection fractions (LVEFs).

**Table 1:** Baseline characteristics of the study cohort by tertiles of the serum galectin-1 concentration.

Characteristic	Tertile 1 (n = 266) Galectin 1 <16.0	Tertile 2 (n = 266) Galectin 1: 16.1–22.2	Tertile 3 (n = 266) Galectin 1 ≥ 22.2	*P*
Age (years)	61 (54–71)	67 (60–75)	73.0 (62–81)	<0.001
Male, n (%)	180 (67.7)	174 (65.4)	185 (69.5)	0.594
Smoking, n (%)	75 (28.2)	91 (34.2)	99 (37.2)	0.080
BMI (kg/m^2^)	24.8 (22.4–27.6)	25.8 (23.8–28.5)	25.7 (22.9–28.2)	0.019
Medical history, *n* (%)				
Hypertension	148 (55.6)	175 (65.8)	199 (74.8)	<0.001
Diabetes	70 (26.3)	86 (32.3)	119 (44.7)	<0.001
Chronic kidney disease	5 (1.9)	5 (1.9)	39 (14.7)	<0.001
Heart failure	9 (3.4)	9 (3.4)	33 (12.4)	<0.001
Peripheral arterial disease	18 (6.8)	12 (4.5)	31 (11.7)	0.007
Previous stroke	12 (4.5)	12 (4.5)	22 (8.3)	0.101
Medications, *n* (%)				
Antiplatelet agents	136 (51.1)	136 (51.1)	152 (57.1)	0.276
ACEi/ARB	61 (22.9)	80 (30.1)	92 (34.6)	0.012
Diuretics	10 (3.8)	26 (9.8)	36 (13.2)	0.001
Oral antidiabetic agents	41 (15.4)	50 (18.8)	57 (21.4)	0.202
Insulin	9 (3.4)	13 (4.9)	23 (8.6)	0.025
Statins	84 (31.6)	90 (33.8)	71 (26.7)	0.189
Laboratory data				
White blood cells (K/cumm)	6.6 (5.5–7.6)	6.7 (5.7–7.8)	6.9 (5.8–7.9)	0.195
Hemoglobin (g/dL)	13.5 (12.5–14.3)	13.4 (12.4–14.2)	12.8 (11.3–14.0)	<0.001
Fasting glucose (mg/dL)	97.5 (87–119)	98.0 (89–114)	101 (91–128)	0.044
HbA1c (%)	6.5 (6.0–7.3)	6.3 (5.8–7.4)	6.5 (5.9–7.3)	0.419
Proteinuria (mg/dL)	0.0 (0.0–0.0)	0.0 (0.0–0.0)	0.0 (0.0–15.0)	<0.001
Proteinuria ≥ 30 mg/dL, n (%)	10 (3.8)	15 (5.7)	53 (20.3)	<0.001
eGFR (ml/min/1.73 m^2^)	80.5 (68–9)	70.7 (59–81)	56.9 (42–73)	<0.001
Total cholesterol (mg/dL)	160 (143–182)	159 (137–182)	159 (137–182)	0.487
Galectin 1 (ng/mL)	13.3 (10.2–14.6)	18.4 (17.3–20.0)	27.7 (24.5–32.6)	<0.001
Cardiac catheterization				
Mean blood pressure (mmHg)	105 (95–113)	105 (92–115)	108 (98–118)	0.281
LVEF (%)	59 (54–64)	60 (54–64)	57 (52–62)	0.002
Significant CAD, n (%)	113 (43)	135 (51)	152 (57)	0.003
Underwent PCI, n (%)	111 (42)	124 (47)	120 (45)	0.509
Contrast volume (ml)	50 (50–95)	50 (50–180)	50 (50–225)	0.002
Outcomes				
CIN, n (%)	7 (2.6)	12 (4.5)	22 (8.3)	0.011
eGFR decline > 30%, n (%)	7 (2.6)	20 (7.5)	53 (19.9)	<0.001
All-cause mortality, n (%)	4 (1.5)	1 (0.4)	8 (3.0)	0.055

BMI, body mass index; ACEi/ARB, angiotensin-converting enzyme inhibitor/angiotensin receptor blocker; HbA1c, glycosylated hemoglobin A1c; eGFR, estimated glomerular filtration rate; LVEF, left ventricular ejection fraction; CAD, coronary artery disease; PCI, percutaneous coronary intervention; CIN, contrast-induced nephropathy.

**Figure 2:**
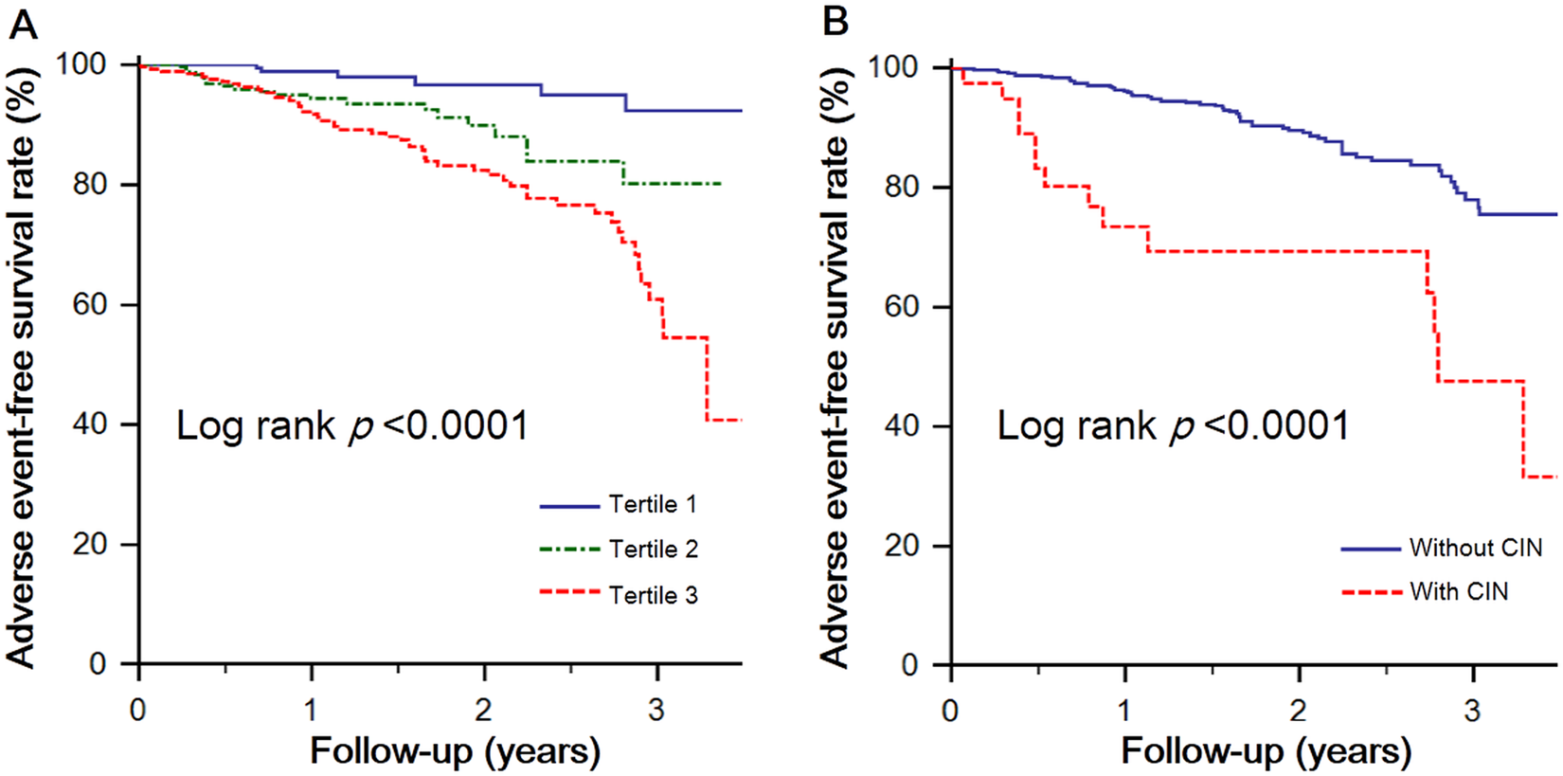
Kaplan–Meier curves of freedom from renal function decline events by **(A)** tertiles of the serum galectin-1 concentration and **(B)** the incidence of contrast-induced nephropathy.

Kaplan–Meier survival analysis was performed to investigate the potential impact of the baseline galectin-1 level on adverse event–free survival (Fig. 2). Patients in the highest galectin-1 group were at significantly greater risk of renal function decline than were those in the lowest galectin-1 group (*p* < 0.001). Patients with prior CIN were also at significantly greater risk of renal function decline than were those without prior CIN (*p* < 0.001).

### Baseline characteristics according to the presence of diabetes

Of the 798 study subjects, 275 (34.5%) were diabetic. Table 2 shows the clinical and demographic characteristics of patients grouped by the presence of diabetes mellitus. Patients with diabetes were older and had higher incidences of hypertension, CKD, and peripheral arterial disease. In addition to anti-diabetic agents, diabetic patients also received more antiplatelet therapy, statins, and anti-hypertensive agents. Compared with nondiabetic patients, patients with diabetes had higher levels of serum galectin-1, more proteinuria, lower hemoglobin and total cholesterol levels, and reduced LVEFs.

**Table 2:** Baseline characteristics of the study cohort according to the presence of diabetes mellitus.

Characteristic	Non-DM (n = 523)	DM (n = 275)	*P*
Age (years)	65 (58–76)	70 (60–78)	0.003
Male, n (%)	344 (65.8)	195 (70.9)	0.152
Smoking, n (%)	168 (32.1)	97 (35.3)	0.385
BMI (kg/m^2^)	25.1 (22.9–27.9)	25.8 (23.4–28.3)	0.039
Medical history, *n* (%)			
Hypertension	296 (56.6)	226 (82.2)	<0.001
Chronic kidney disease	14 (2.7)	35 (12.7)	<0.001
Heart failure	28 (5.4)	23 (8.4)	0.127
Peripheral arterial disease	20 (3.8)	41 (15.0)	<0.001
Previous stroke	29 (5.6)	17 (6.2)	0.750
Medications, *n* (%)			
Antiplatelet agents	254 (48.6)	170 (61.8)	<0.001
ACEi/ARB	128 (24.5)	105 (38.2)	<0.001
Diuretics	35 (6.7)	36 (13.1)	0.004
Oral antidiabetic agents	0 (0.0)	148 (53.8)	<0.001
Insulin	0 (0.0)	45 (16.4)	<0.001
Statins	144 (27.5)	101 (36.7)	0.010
Laboratory data			
White blood cells (K/cumm)	6.6 (5.6–7.6)	7.0 (5.7–8.2)	0.007
Hemoglobin (g/dL)	13.4 (12.4–14.3)	12.7 (11.3–13.8)	<0.001
Fasting glucose (mg/dL)	94 (87–102)	123 (103–153)	<0.001
HbA1c (%)	5.9 (5.6–6.1)	7.1 (6.5–8.0)	<0.001
Proteinuria (mg/dL)	0.0 (0.0–0.0)	0.0 (0.0–15.0)	<0.001
Proteinruia ≥ 30 mg/dL, n (%)	24 (2.7)	54 (19.9)	<0.001
eGFR (ml/min/1.73 m^2^)	72.7 (59.8–85.7)	64.5 (44.8–80.8)	<0.001
Total cholesterol (mg/dL)	165 (143–186)	152 (132–175)	<0.001
Galectin 1 (ng/ml)	17.0 (14.2–23.0)	20.5 (15.9–28.5)	<0.001
Cardiac catheterization			
Mean blood pressure (mmHg)	104 (93–112)	109.5 (98–122)	0.003
LV ejection fraction (%)	59.0 (54.0–64.0)	56.8 (52.0–62.0)	0.014
Significant CAD, n (%)	244 (46.7)	156 (56.7)	0.007
Underwent PCI, n (%)	210 (40.2)	145 (52.7)	0.001
Contrast volume (ml)	50.0 (50.0–120.0)	60.0 (50.0–195.0)	<0.001
Outcomes			
CIN, n (%)	18 (3.4)	23 (8.4)	0.004
eGFR decline > 30%, n (%)	36 (6.9)	44 (16.0)	<0.001
All-cause mortality, n (%)	7 (1.3)	6 (2.2)	0.388

DM, diabetes mellitus; BMI, body mass index; ACEi/ARB, angiotensin-converting enzyme inhibitor/angiotensin receptor blocker; HbA1c, glycosylated hemoglobin A1c; eGFR, estimated glomerular filtration rate; LV, left ventricular; CAD, coronary artery disease; PCI, percutaneous coronary intervention; CIN, contrast-induced nephropathy.

### Independent correlates of contrast-induced nephropathy and predictors of renal function decline

In univariate logistic regression analysis, a higher galectin-1 level, older age, history of diabetes, lower hemoglobin level, decreased baseline eGFR, and presence of proteinuria were associated significantly with a greater risk of CIN. To identify independent predictors of CIN, multivariable logistic regression analysis was performed. After adjustment for age, sex, and baseline eGFR, the highest serum galectin-1 level was associated significantly with CIN [odds ratio (OR), 2.64; 95% confidence interval (CI), 1.03–6.76; *p* = 0.04]. After adjustment for age, sex, and all factors that were significant in the univariate analysis, the serum galectin-1 level was not associated significantly with CIN, but associations persisted with age (OR, 1.04; 95% CI, 1.01–1.08; *p* = 0.026), and the hemoglobin level (OR, 0.67; 95% CI, 0.54–0.83; *p* < 0.001; Table 3).

**Table 3:** Multivariable logistic regression analysis of the association between the serum galectin-1 concentration and the incidence of contrast-induced nephropathy.

Outcome	Univariable		Model 1*		Model 2^†^	
	Crude OR (95% CI)	*P*	Adjusted OR (95% CI)	*P*	Adjusted OR (95% CI)	*P*
Galectin-1						
* tertile 1*	*Reference*		*Reference*		*Reference*	
* tertile 2*	1.75 (0.68–4.51)	0.248	1.54 (0.59–4.02)	0.381	1.80 (0.67–4.81)	0.244
* tertile 3*	3.34 (1.40–7.95)	0.007	2.64 (1.03–6.76)	0.044	2.04 (0.77–5.43)	0.152
Age	1.04 (1.01–1.07)	0.003	1.04 (1.00–1.07)	0.034	1.04 (1.01–1.08)	0.026
Gender	0.74 (0.39–1.41)	0.358	0.68 (0.35–1.31)	0.250	0.86 (0.42–1.76)	0.674
HTN	1.68 (0.81–3.48)	0.163				
Diabetes	2.56 (1.36–4.83)	0.004			1.82 (0.89–3.70)	0.101
Hb	0.63 (0.53–0.75)	<0.001			0.67 (0.54–0.83)	<0.001
eGFR	0.98 (0.97–1.00)	0.033	1.00 (0.98–1.02)	0.853	1.02 (1.00–1.04)	0.062
Proteinuria	1.01 (1.00–1.01)	0.004			1.00 (1.00–1.01)	0.057
LVEF	0.99 (0.96–1.02)	0.457				
Contrast	1.00 (1.00–1.01)	0.150				

OR, odds ratio; CI, confidence interval; HTN, hypertension; Hb, hemoglobin; eGFR, estimated glomerular filtration rate; LVEF, left ventricular ejection fraction.

*Adjusted for age, sex, and baseline eGFR; ^†^adjusted for age, sex, and variables with *p* values < 0.05 in the univariable analysis.

In a univariate Cox regression analysis, older age, histories of hypertension and diabetes, proteinuria, higher serum galectin-1 level, lower hemoglobin level, reduced eGFR, and lower LVEF, as well as prior CIN, were associated significantly with a higher incidence of renal function decline. In a multivariable Cox regression analysis, the circulating galectin-1 level [tertile 2: hazard ratio (HR) 5.56, 95% CI 1.79–17.22; tertile 3: HR 5.67, 95% CI 1.97–16.32), hemoglobin level (HR, 0.83; 95% CI, 0.71–0.98; *p* = 0.025) and proteinuria (HR, 1.00; 95% CI, 1.00–1.01; *p* = 0.006) remained associated significantly with renal function decline (Table 4). Prior CIN was associated with renal function decline at a borderline significance level (HR, 1.98; 95% CI, 0.96–4.09; *p* = 0.063). The serum galectin-1 level was an independent predictor of renal function decline, rather than CIN, in patients undergoing CAG.

**Table 4:** Multivariable Cox proportional-hazard analysis of the association of the serum galectin-1 concentration with the incidence of renal function decline.

Outcome	Univariable		Model 1*		Model 2^†^	
	Crude HR (95% CI)	*P*	Adjusted HR (95% CI)	*P*	Adjusted HR (95% CI)	*P*
Galectin-1						
* tertile 1*	*Reference*		*Reference*		*Reference*	
* tertile 2*	4.23 (1.63–10.99)	0.003	4.13 (1.59–10.73)	0.004	5.56 (1.79–17.22)	0.003
* tertile 3*	8.22 (3.38–19.97)	<0.001	6.62 (2.67–16.40)	<0.001	5.67 (1.97–16.32)	0.001
Age	1.02 (1.00–1.04)	0.019	1.01 (0.99–1.03)	0.585	1.01 (0.99–1.04)	0.343
Gender	1.00 (0.63–1.60)	0.997	0.98 (0.61–1.57)	0.926	0.91 (0.53–1.58)	0.746
HTN	2.61 (1.46–4.64)	0.001			1.61 (0.81–3.19)	0.172
Diabetes	2.43 (1.56–3.78)	<0.001			1.31 (0.75–2.31)	0.343
Hb	0.75 (0.66–0.85)	<0.001			0.83 (0.71–0.98)	0.025
eGFR	0.98 (0.97–0.99)	<0.001	0.99 (0.98–1.01)	0.077	0.99 (0.98–1.01)	0.400
Proteinuria	1.01 (1.00–1.01)	<0.001			1.00 (1.00–1.01)	0.006
LVEF	0.96 (0.94–0.98)	<0.001			0.98 (0.96–1.00)	0.070
Prior CIN	4.01 (2.25–7.15)	<0.001			1.98 (0.96–4.09)	0.063

HR, hazard ratio; CI, confidence interval; HTN, hypertension; Hb, hemoglobin; eGFR, estimated glomerular filtration rate; LVEF, left ventricular ejection fraction; CIN, contrast-induced nephropathy.

*Adjusted for age, sex, and baseline eGFR; ^†^adjusted for age, sex, and variables with *p* values < 0.05 in the univariable analysis.

### Subgroup analysis

The study cohort was stratified according to the presence of diabetes, presence of proteinuria, and PCI status. Increasing galectin-1 concentrations were associated significantly with renal function decline, irrespective of underlying diseases, such as diabetes and proteinuria, or of PCI status (*p*_interaction_ > 0.05; Table 5).

**Table 5:** Stratified analysis of the effect of the serum galectin-1 concentration on renal function decline in patients grouped by diabetes, proteinuria, and percutaneous coronary intervention status.

Subgroup (events/subjects)	Galectin-1		*P* for interaction
	Adjusted HR (95% CI)	*P*	
**Overall** (80/798)			
*tertile 2*	5.56 (1.79–17.22)	0.003	
*tertile 3*	5.67 (1.97–16.32)	0.001	
**With diabetes** (44/275)			
*tertile 2*	3.88 (0.92–16.41)	0.065	0.236
*tertile 3*	3.55 (0.99–12.81)	0.053	
**Without diabetes** (36/523)			
*tertile 2*	10.45 (1.27–85.77)	0.029	
*tertile 3*	12.11 (1.57–93.40)	0.017	
**With proteinuria** (42/78)			
*tertile 2*	7.57 (0.76–75.56)	0.085	0.560
*tertile 3*	9.61 (1.17–79.11)	0.035	
**Without proteinuria** (38/720)			
*tertile 2*	3.90 (1.04–14.62)	0.043	
*tertile 3*	3.72 (1.07–13.02)	0.039	
**Underwent PCI** (39/355)			
*tertile 2*	9.32 (1.12–77.44)	0.039	0.115
*tertile 3*	8.51 (1.06–68.23)	0.044	
**Not underwent PCI** (41/443)			
*tertile 2*	2.08 (0.38–11.52)	0.400	
*tertile 3*	3.13 (0.84–11.67)	0.089	

HR, hazard ratio; CI, confidence interval; PCI: percutaneous coronary intervention.

*Adjusted for age, sex, hypertension, diabetes, hemoglobin, baseline estimated glomerular filtration rate, proteinuria, left ventricular ejection fraction, and prior contrast-induced nephropathy.

## Discussion

In this single-center observational study, the serum galectin-1 concentration was associated with eGFR decline during a mean follow-up period of 1.4 ± 1.1 years in 798 patients with stable angina undergoing elective CAG, irrespective of the presence of diabetes. To our knowledge, this longitudinal study is the first to explore the relationship between galectin-1 and subsequent renal function deterioration in patients undergoing CAG. These results demonstrate the involvement of galectin-1 in chronic renal function impairment, and suggest that the circulating galectin-1 level is an independent predictor of chronic renal function decline.

Much research attention has focused on galectin-1 and galectin-3, given their apparent major roles in cancer biology^23^. Recent reviews and meta-analyses have demonstrated that galectin-1 is associated with tumor formation, progression, metastasis, angiogenesis, and prognosis in several kinds of cancer^23,24^. Intracellularly, the galectin-1 protein provides a carbohydrate-independent scaffold for intracellular signaling pathways; extracellularly, it governs β-galactoside binding. Multivalent interactions between galectin-1 and glycoproteins in the extracellular matrix contribute to cancer metastasis. A few *in vitro* and animal studies have mentioned the potential link between galectin-1 and kidney diseases^20–22,25–28^, but no previous clinical report has described an association between the galectin-1 level and CKD.

The mechanisms linking galectin-1 elevation to CKD progression are not fully clear. Several groups have postulated the existence of mechanisms relating galectin-1 to CKD^20^, especially in the context of diabetes^21,22^. Increased galectin-1 expression has been reported in diabetes^19^, diabetic retinopathy^29^ and diabetic nephropathy^21,22^. Galectin-1 has been shown to regulate podocin production and damage, and diabetic nephropathy progression, in podocytes^22^. In addition, recent evidence has revealed that galectin-1 is a fibrosis protein that is highly expressed in the kidneys of mice with types 1 and 2 diabetes, and activated in proximal tubular epithelial cells under high-glucose conditions^22^. The phosphorylation and activation of Akt also may play crucial roles in the modulation of activating enhancer binding protein 4 (AP4) to up-regulate the galectin-1 protein under hyperglycemic conditions in diabetic mice^22^. AP4 was identified as a protein binding to the galectin-1 promoter that regulates various functions under high glucose stimulation^22^.

Less is known about the association between galectin-1 and CKD in non-diabetic contexts. Emerging evidence suggests that the progression of cardiac and renal functions is often interconnected^30,31^. Cardiac and renal diseases have several common pathways, including those of enhanced systemic inflammation and stress-mediated neurohormonal responses, the development of anemia and bone and mineral disorders, and acid–base and fluid imbalances^31^. Chronic myocardial dysfunction is also a well-known mediator of progressive renal function decline. The pathophysiology of this so-called type 2 cardiorenal syndrome remains under investigation^30^. Interestingly, a recent review suggested that galectin-1 is an emergent mediator of cardiovascular inflammation^32^. In this study, we also found that galectin-1 was associated with the LVEF and CAD in univariate analysis. We speculate that the parallel process affecting both heart and kidney functions also plays an essential role in the non-diabetic association between galectin-1 and CKD. Our findings, in agreement with previous reports, illustrate the association of galectin-1 with renal function decline irrespective of diabetes. Galectin-1 thus may play a critical role in renal function decline in diabetic and non-diabetic patients.

Interestingly, we found no significant association between galectin-1 and CIN in patients undergoing CAG or PCI procedures. One possible explanation is that the mechanisms of CIN differ from those of chronic renal decline. Contrast media may alter nitric oxide, endothelin, and adenosine levels, inducing renal vasoconstriction and leading to renal medulla ischemia and acute tubular necrosis^33,34^. Contrast media may also have cytotoxic effects via the upregulation of reactive oxygen species^33,35^ or direct induction of osmotic tubular nephrosis^36^. Another possible reason is the lower incidence of CIN than renal function decline in our study sample. Furthermore, galectin-1 and diabetes were correlated in this study and in previous reports^17,18^. Before adjustment for diabetes, tertile 3 of the galectin-1 level correlated with CIN. After adjustment for all related risk factors, including diabetes, this association was insignificant.

This study has some limitations that should be considered. First, the study population was relatively small, and consisted of Asian patients treated at a single hospital. Further studies larger numbers of different participants are required to confirm our findings. Second, patients enrolled in our study were elders (mean age, 67 ± 12 years). Caution should be taken when applying our findings to younger populations. Finally, the eGFR was used as the sole renal function endpoint, without consideration of other clinical endpoints affecting renal outcomes, including newly diagnosed diabetes mellitus and the progression of proteinuria. Nevertheless, our study demonstrated that the serum galectin-1 level is a novel risk marker for renal outcomes in patients with suspected CAD undergoing CAG or PCI procedures.

## Conclusions

Although not a predictor of CIN, the circulating serum galectin-1 level is an independent prognostic marker for subsequent renal function decline in patients undergoing CAG, irrespective of diabetes. These findings provide novel evidence of galectin-1’s involvement in the pathogenesis of renal dysfunction in patients with suspected CAD. Further research exploring the underlying mechanism is needed.

## Methods

### Study design and patient population

A team of cardiologists (under the direction of Prof. Po-Hsun Huang) collected data for this study consecutively from June 2011 to August 2018. The sample size was not pre-calculated. In total, 855 subjects with stable coronary CAD admitted to Taipei Veterans General Hospital for elective CAG and/or PCI were screened. Each patient’s serum creatinine concentration was measured before CAG, and the eGFR was calculated using the CKD Epidemiology Collaboration equation^37^. Patients with stage 5 CKD, defined as creatinine clearance < 15 ml/min/1.73 m^2^ (*n* = 15), and those undergoing hemodialysis or peritoneal dialysis (*n* = 42) were excluded from the analysis. Thus, a total of 798 patients was enrolled in the study and included in the final analysis (Fig. 1). The patients were grouped into tertiles according to the serum galectin-1 concentration.

Each patient’s chart was reviewed in detail to collect data on medications, smoking status, and risk factors for CIN, such as age, existing renal dysfunction, type 2 diabetes mellitus, and volume depletion. Patients’ blood pressure was measured using electronic sphygmomanometers at least four times a day during hospitalization. Hypertension was defined as systolic blood pressure ≥ 140 mmHg, diastolic blood pressure ≥ 90 mmHg, or the use of antihypertensive medications. Type 2 diabetes mellitus was defined as fasting plasma glucose level ≥ 126 mg/dl or the use of hypoglycemic agents. The body mass index was calculated by dividing the weight of the patient (in kilograms) by the square of the height (in meters). A nonionic low-osmolality contrast medium (iopromide) was used for all patients’ examinations. It was administered intra-arterially, mainly through transradial catheters. Metformin and nephrotoxic medications, such as non-steroidal anti-inflammatory drugs, were discontinued 48 h before contrast medium administration. Before and after contrast medium exposure, physiological (0.9%) saline was given intravenously at a rate of 1 ml/kg/h for 12 h. In patients with left ventricular dysfunction (ejection fraction < 40%) or apparent heart failure, the hydration rate was reduced to 0.5 ml/kg/h. This research was conducted according to the principles expressed in the Declaration of Helsinki. This study was approved by the research ethics committee of Taipei Veterans General Hospital, and all participants provided written informed consent.

### Laboratory investigations and cardiac catheterization

Blood samples were collected after an ≥ 8-h fast. The blood cell count; serum glucose, creatinine, and uric acid levels; and lipid profiles were determined using a Hitachi 7600 autoanalyzer (Hitachi Ltd., Tokyo, Japan). The serum creatinine concentration was assessed at the time of admission and daily for the following 3 days after contrast medium exposure. Urine dipstick analysis was performed using commercial test strips, and proteinuria was defined as a urine protein concentration ≥ 30 mg/100 ml. Plasma concentrations of galectin-1 were determined using the commercially available Human Galectin-1 Quantikine ELISA Kit DGAL10 (R&D Systems, Inc., Minneapolis, MN, USA); the sensitivity was 0.129 ng/ml and the assay range was 0.3–20 ng/ml. The intra- and interassay coefficients were 5.7–8.8% and 7.5–9.5%, respectively. Two experienced interventional cardiologists interpreted the coronary angiograms. Coronary lesions causing > 50% diameter narrowing were considered to represent significant stenosis. The LVEF was estimated by left ventriculography. The contrast consumption of each patient was also recorded.

### Definition of study endpoints and renal function decline

All patients were evaluated for the occurrence of CIN, which was defined as an increase in the serum creatinine concentration of ≥0.5 mg/dl or a 25% increase from baseline within 48 h after CAG^38^. Patients were advised to visit outpatient clinics regularly after discharge from the hospital. The cohort was followed until January 2019 or the occurrence of death. Patients’ clinical data, including serum creatinine levels, were obtained every 3–6 months during the follow-up period. Renal function decline was defined as a > 30% decrease in the eGFR after discharge^39,40^. When it was identified, the serum creatinine level was re-measured 1 month later to ensure the accuracy of the diagnosis.

### Statistical analysis

Data were expressed as medians (interquartile ranges) for numeric variables and as numbers (percentages) for categorical variables. Clinical and laboratory data were compared using the Mann–Whitney U test for continuous variables and Fisher’s exact test for categorical variables. The incidences of CIN and renal function decline were calculated. Survival curves were generated using the Kaplan–Meier method, and survival was compared among groups using the log-rank test. Logistic regression analysis was performed to examine the relationships of various risk factors to CIN, and Cox proportional-hazard regression analysis was performed to identify risk factors for progressive renal function decline. Factors that were significant in univariate regression analysis were entered into the multivariable regression analysis. To investigate modification of the effect of galectin-1 on renal function decline by different comorbidities, we performed subgroup analyses with the study cohort stratified according to the presence of diabetes, presence of proteinuria, and PCI status. Interactions between the galectin-1 concentration and comorbid conditions were examined by adding a product term of galectin-1 and comorbidities to the Cox regression analysis. Data were analyzed using SPSS version 18.0 (SPSS Inc., Chicago, IL). P values < 0.05 were regarded as significant.

### Data availability

The datasets generated and/or analyzed during the current study are available from the corresponding author on reasonable request.
